# Synthetic Denitrifying Communities Reveal a Positive and Dynamic Biodiversity-Ecosystem Functioning Relationship during Experimental Evolution

**DOI:** 10.1128/spectrum.04528-22

**Published:** 2023-05-08

**Authors:** Bo Wu, Xiaotong Guan, Ting Deng, Xueqin Yang, Juan Li, Min Zhou, Cheng Wang, Shanquan Wang, Qingyun Yan, Longfei Shu, Qiang He, Zhili He

**Affiliations:** a Environmental Microbiomics Research Center, School of Environmental Science and Engineering, Southern Marine Science and Engineering Guangdong Laboratory (Zhuhai), Sun Yat-sen University, Guangzhou, China; b College of Agronomy, Hunan Agricultural University, Changsha, China; c Department of Civil and Environmental Engineering, The University of Tennessee, Knoxville, Tennessee, USA; University of Nebraska—Lincoln

**Keywords:** *Shewanella*, synthetic microbial community, biodiversity-ecosystem functioning relationship, complementarity effect, experimental evolution, selection effect

## Abstract

Biodiversity is vital for ecosystem functions and services, and many studies have reported positive, negative, or neutral biodiversity-ecosystem functioning (BEF) relationships in plant and animal systems. However, if the BEF relationship exists and how it evolves remains elusive in microbial systems. Here, we selected 12 *Shewanella* denitrifiers to construct synthetic denitrifying communities (SDCs) with a richness gradient spanning 1 to 12 species, which were subjected to approximately 180 days (with 60 transfers) of experimental evolution with generational changes in community functions continuously tracked. A significant positive correlation was observed between community richness and functions, represented by productivity (biomass) and denitrification rate, however, such a positive correlation was transient, only significant in earlier days (0 to 60) during the evolution experiment (180 days). Also, we found that community functions generally increased throughout the evolution experiment. Furthermore, microbial community functions with lower richness exhibited greater increases than those with higher richness. Biodiversity effect analysis revealed positive BEF relationships largely attributable to complementary effects, which were more pronounced in communities with lower richness than those with higher richness. This study is one of the first studies that advances our understanding of BEF relationships and their evolutionary mechanisms in microbial systems, highlighting the crucial role of evolution in predicting the BEF relationship in microbial systems.

**IMPORTANCE** Despite the consensus that biodiversity supports ecosystem functioning, not all experimental models of macro-organisms support this notion with positive, negative, or neutral biodiversity-ecosystem functioning (BEF) relationships reported. The fast-growing, metabolically versatile, and easy manipulation nature of microbial communities allows us to explore well the BEF relationship and further interrogate if the BEF relationship remains constant during long-term community evolution. Here, we constructed multiple synthetic denitrifying communities (SDCs) by randomly selecting species from a candidate pool of 12 *Shewanella* denitrifiers. These SDCs differ in species richness, spanning 1 to 12 species, and were monitored continuously for community functional shifts during approximately 180-day parallel cultivation. We demonstrated that the BEF relationship was dynamic with initially (day 0 to 60) greater productivity and denitrification among SDCs of higher richness. However, such pattern was reversed thereafter with greater productivity and denitrification increments in lower-richness SDCs, likely due to a greater accumulation of beneficial mutations during the experimental evolution.

## INTRODUCTION

Biodiversity is vital for ecosystem functioning that supplies essential goods and services to all species on the planet (1, 2). Previous studies of plant and animal systems have shown that diverse communities were more productive (3) and more stable (4, 5). Thus, biodiversity is considered a primary factor that moderates ecosystem functions (6–8). Microorganisms play important roles in all ecosystems, such as the global biogeochemical cycling of nutrients (9, 10), regulation of greenhouse gas (e.g., CO_2_, CH_4_, N_2_O) emissions (11–13), bioremediation of eutrophication and contaminated environments (14, 15), and the health improvement of human (16). Therefore, understanding biodiversity-ecosystem function (BEF) relationships is crucial for us to predict microbially driven ecological processes as well as ecosystem services in microbial ecosystems.

In natural environments, positive BEF relationships between biodiversity and ecosystem productivity were observed and explained by complementarity and selection effects (17). The complementarity effect refers to niche differentiation or positive interactions that improve resource utilization and increase the productivity of ecosystems (18–20), while the selection effect refers to the dominance by species with particular traits that affect community composition and diversity (21, 22). Although positive BEF relationships were commonly observed, negative (23) and neutral (24, 25) BEFs were also revealed by manipulating microbial diversity levels, such as natural gradients (26) and serial dilutions (27, 28). However, studying natural microbial communities could be hindered by the inability to control environmental conditions, a lack of adequate knowledge about the ecophysiology of diverse species, or populations that are frequently poorly characterized in natural environments.

Recently, considerable attention has been paid to synthetic microbial communities with the features of low complexity and high controllability, which could serve as model systems to improve our understanding of fundamental ecological principles and theories (29–31), such as BEF relationships (32, 33), biodiversity-stability relationships (34), and niche differentiation (35). For example, it was reported that diverse communities generally had high productivity with synthetic microbial communities under abiotic perturbations (32). Therefore, synthetic microbial communities may be a powerful tool to reveal BEF relationships, their dynamics, and underlying mechanisms.

Currently, microbial BEF studies were only conducted at a single time point or for a short time period, but evolutionary events of microbial communities could have profound effects on ecosystem functioning (18). Microbial experimental evolution uses microorganisms with well-defined physiological and genomic backgrounds to understand evolutionary adaptation and underlying mechanisms, bringing great power and precision to the study of microbial ecology and evolutionary biology (36, 37). For example, this approach has been used to explore mechanisms of evolution, such as stress adaptation and genomic dynamics in Escherichia coli, Desulfovibrio vulgaris, and Saccharomyces cerevisiae (38–41). Also, growing evidence showed that evolutionary dynamics could constrain BEF relationships (42–45). For example, a stronger evolutionary response in low-diversity microbial communities after an approximately 5-month evolution was observed. Individual focal strains have been added in complex communities, and genetic changes involved in carbon metabolisms and interspecific interactions were detected, demonstrating the influence of evolution on BEF relationships (42). Therefore, it is necessary to explore BEF relationships using synthetic microbial communities with defined physiological and genomic backgrounds and under an evolution experiment.

In this study, we aimed to understand the BEF relationship of microbial communities and their intergenerational dynamics. The metabolic versatility of *Shewanella* species makes them ubiquitous in diverse environments, and they are important denitrifiers since all known *Shewanella* species could reduce nitrate using a variety of different electron donors (46). Particularly, their well-annotated genomes could facilitate our further understanding of molecular mechanisms for such observed biodiversity-ecosystem functioning relationships (47). We hypothesized that microbial richness would be positively correlated with ecosystem functioning (e.g., productivity, denitrification) due to complementarity effects (20) and selection effects (48), and such BEF relationships would be dynamic as microbial species interactions could be constrained during experimental evolution (42–44). To test these hypotheses, we selected 12 *Shewanella* denitrifiers to construct synthetic denitrifying communities (SDCs) at the richness level of 1, 2, 4, 8 and 12 species and analyzed their growth, denitrification rate, interaction and dynamics over the course of experimental evolution. We found positive and dynamic BEF relationships of SDCs during the evolution experiment. This study advances our understanding of BEF relationships of microbial communities and their possible dynamic mechanisms, which has important implications for predicting BEF relationships in microbial systems.

## RESULTS

### Relationships between biodiversity and ecosystem functioning.

**FIG 1:**
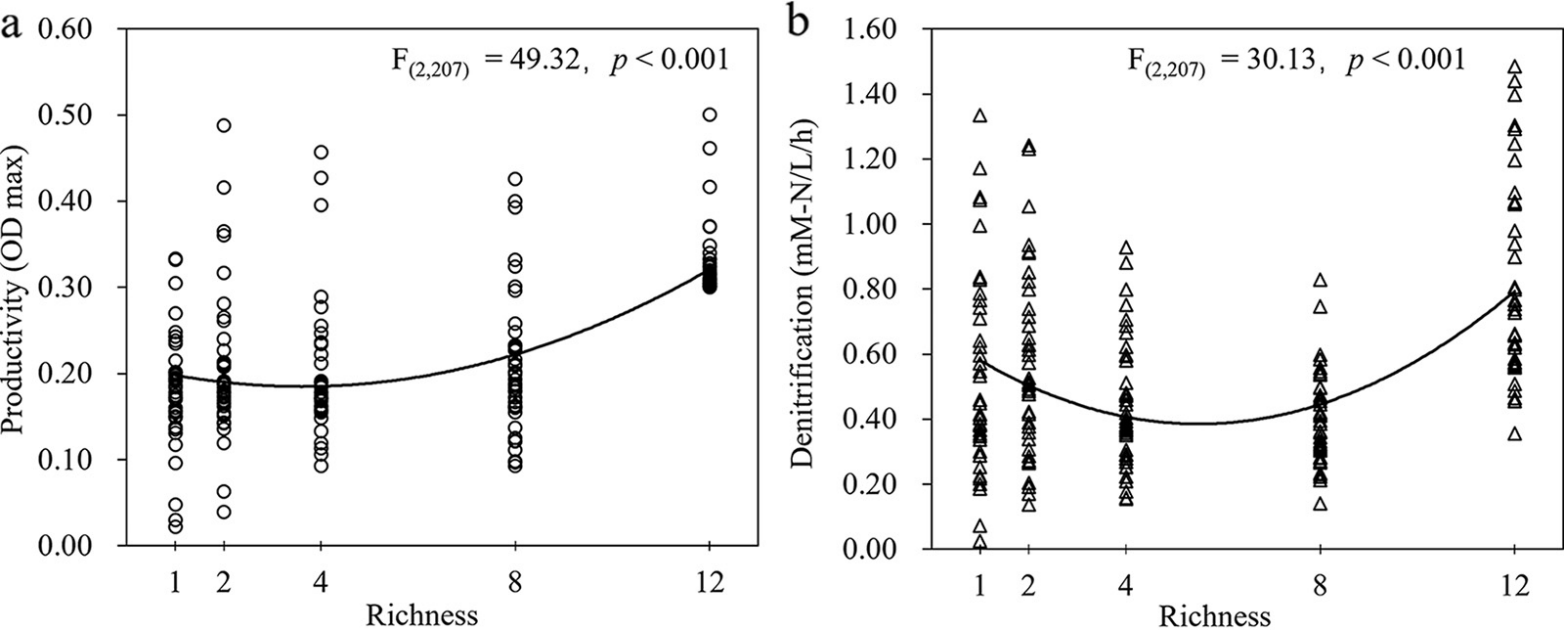
The overall relationship between the community function and species richness of synthetic denitrifying communities (SDCs). (a) A significant positive relationship was found for species richness and productivity (OD_600_) using Spearman’s correlation. (b) A significant positive relationship was also observed between species richness and denitrification using Spearman’s correlation.

The selected 12 *Shewanella* species/strains were cultivated in a modified 2216 medium (see Fig. S1 in the supplemental material) to construct synthetic denitrifying communities (SDCs), and we evolved all SDCs for 180 days. Our results showed that the overall productivity (ρ = 0.444; *P *< 0.001) or denitrification rate (ρ = 0.192; *P* = 0.005) was significantly and positively correlated with the species richness in all SDCs (Fig. 1a and b).

**FIG 2:**
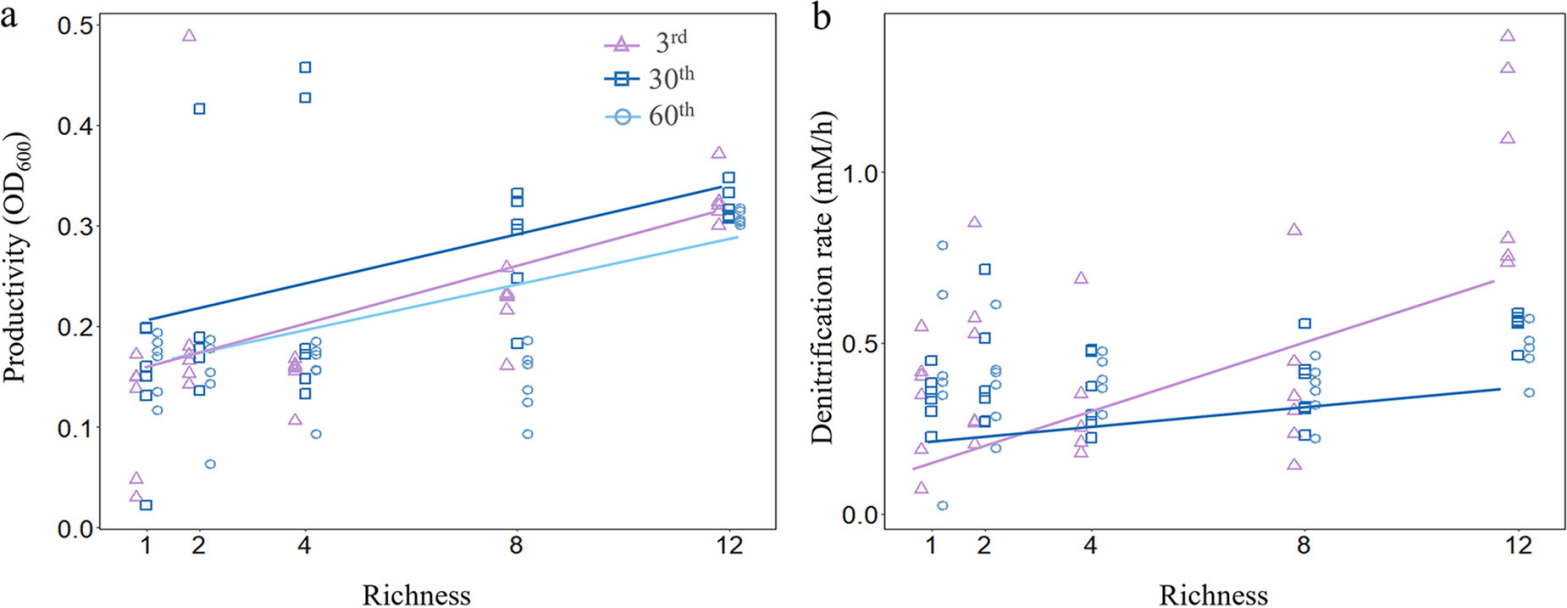
Relationships between species richness and the community function along the evolution experiment. (a) Significant positive linear relationships were found between richness and productivity (OD_600_) at the 3rd day [slope = 0.015; *F*_(1, 28)_ = 20.70; *P* < 0.001], 30th day [slope = 0.014; *F*_(1, 28)_ = 12.04; *P = *0.002], and 60th day [slope = 0.012; *F*_(1, 28)_ = 24.55; *P* < 0.001]. (b) Relationships between species richness and denitrification rate were significantly positive at the 3rd day [slope = 0.051; *F*_(1, 28)_ = 17.68; *P* < 0.001] and 30th day [slope = 0.015; *F*_(1, 28)_ = 7.81; *P = *0.009].

The richness-community function (productivity and denitrification) relationship was dynamic over time during the evolution experiment; productivity was significantly (ρ = 0.737; *P* < 0.001) and positively correlated with the SDC richness at the 3rd (after the first transfer), 30th (ρ = 0.603; *P* < 0.001), and 60th (ρ = 0.388; *P *= 0.034) days (Fig. 2a), but there was not a correlation between productivity and richness detected (*P* > 0.05) at the 90th, 120th, 150th, and 180th days. For denitrification, a significantly positive relationship was found at the 3rd (ρ = 0.433; *P* = 0.017) and 30th days (ρ = 0.444; *P* = 0.014) (Fig. 2b), but no association between denitrification rate and richness was detected (*P* > 0.05) at the 60th, 90th, 120th, 150^th^, and 180th days (Fig. 2b). We also examined the change of SDC functions along the evolution experiment and found that both overall productivity (ρ = 0.227; *P* = 0.001) and denitrification rates (ρ = 0.364; *P* < 0.001) increased significantly along the evolution experiment in all SDCs (see Fig. S2a and b in the supplemental material), but increased rates were higher at low richness SDCs than at high richness SDCs, resulting in a significantly negative relationship between richness and the increase rate of functions expressed as the slope of function versus time for both productivity (ρ = −0.591; *P* < 0.001) and denitrification (ρ = −0.430; *P* = 0.018) (Fig. S2c to f).

### The composition and dynamics of synthetic denitrifying communities over time during the evolution experiment.

**FIG 3:**
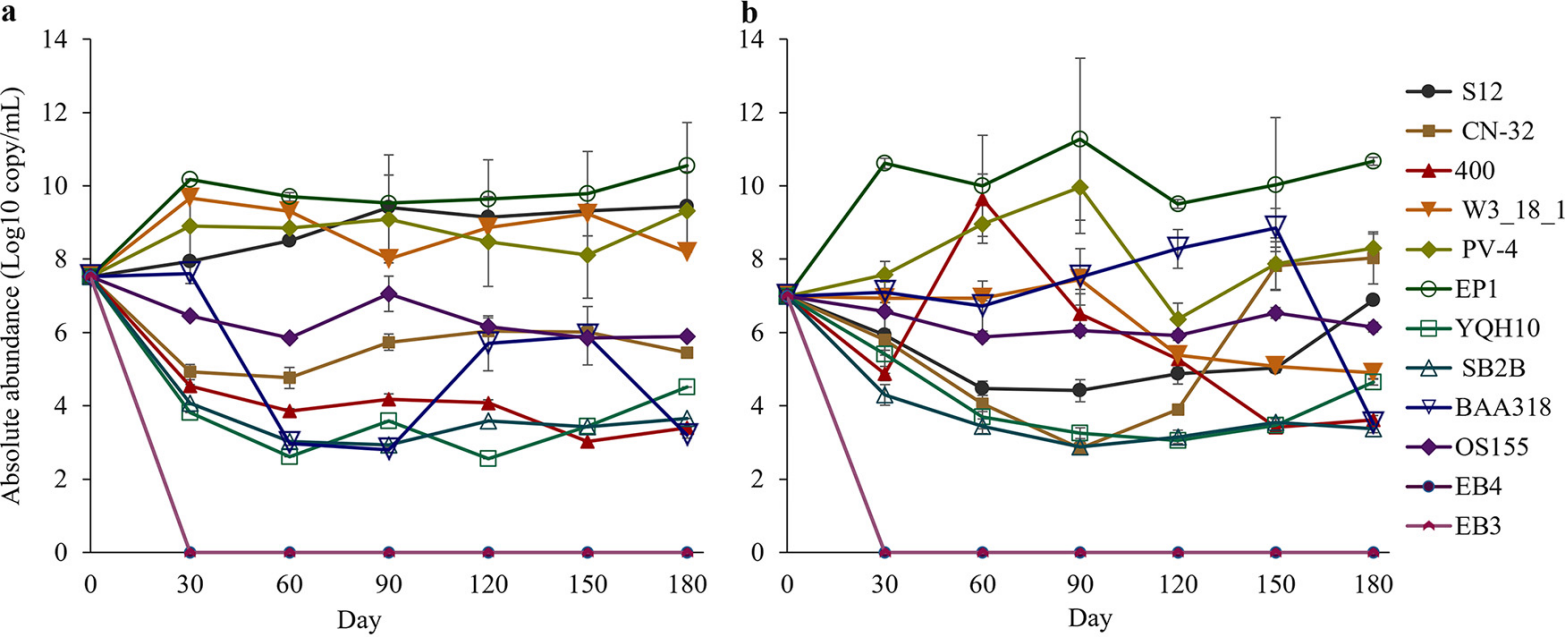
The temporal dynamics of synthetic denitrifying community composition during the evolution experiment. Log-transferred absolute abundances of individual *Shewanella* species/strains in SDCs were shown as mean ± standard deviation (SD) (*n* = 6). (a) Four-species SDCs. (b) Twelve-species SDCs. Species abbreviations are as follows: S12, S. decolorationis S12; CN-32, S. putrefaciens CN-32; 400, S. frigidimarina NCIMB 400; W3_18_1, *Shewanella* sp. W3_18_1; PV-4, *S. loihica* PV-4; EP1, *S. marisflavi* EP1; YQH10, *S. mangrovi* YQH10; SB2B, *S. amazonensis* SB2B; BAA-318, *S. fidelis* ATCC BAA-318; OS155, S. baltica OS155; HAW-EB3, *S. sediminis* HAW-EB3; HAW-EB4, *S. halifaxensis* HAW-EB4.

To understand the composition and dynamics of SDCs, we tracked their species abundances over time during the evolution experiment (Fig. 3; see also Fig. S3 in the supplemental material). The abundance of SDCs fluctuated and generally increased over time during the evolution experiment except in the following four SDCs: 1-3, 4-2, 4-4, and 4-6 (Fig. S3).

Among all SDCs, Shewanella sediminis HAW-EB3 and Shewanella halifaxensis HAW-EB4 were not detected at the 30th day or later, while Shewanella frigidimarina NCIMB 400 was not detected at the 150th day or later of the evolution experiment in some SDCs (e.g., some of 4-, 8-, and 12-species SDCs). However, a general pattern of species abundance was observed. For 4-species SDCs on the 180th day, the species Shewanella marisflavi EP1 (87.5% on the average) and Shewanella loihica PV-4 (5.23% on the average) were the most abundant individuals in different SDCs (Fig. 3a). For 12-species SDCs on the 180th day, the results showed a decrease in abundances in the following order: *S. marisflavi* EP1 (99.3%), *S. loihica* PV-4 (0.428%), and Shewanella putrefaciens CN-32 (0.230%) were the dominant species, followed by Shewanella decolorationis S12 (0.016%), Shewanella baltica OS155 (0.003%), *Shewanella* sp. W3_18_1 (<0.001%), Shewanella mangrovi YQH10 (<0.001%), S. frigidimarina NCIMB 400 (<0.001%), Shewanella fidelis ATCC BAA-318 (<0.001%), and Shewanella amazonensis SB2B (<0.001%) (Fig. 3b). Such patterns of SDCs were generally consistent among different replications at each richness level (see Fig. S3).

To identify the contribution of each species to observed BEF relationships, we analyzed the abundance of all tested species at different richness levels and time. The results showed that the overall relative abundance of *S. marisflavi* EP1 and *S. loihica* PV-4 was higher than their expected yields at each richness level, while the abundance of other species was lower than their expected abundances (see Fig. S4 in the supplemental material), suggesting that *S. marisflavi* EP1 and *S. loihica* PV-4 could play a key role in determining the observed positive BEF relationship.

### Biodiversity effects during the evolution experiment.

**FIG 4:**
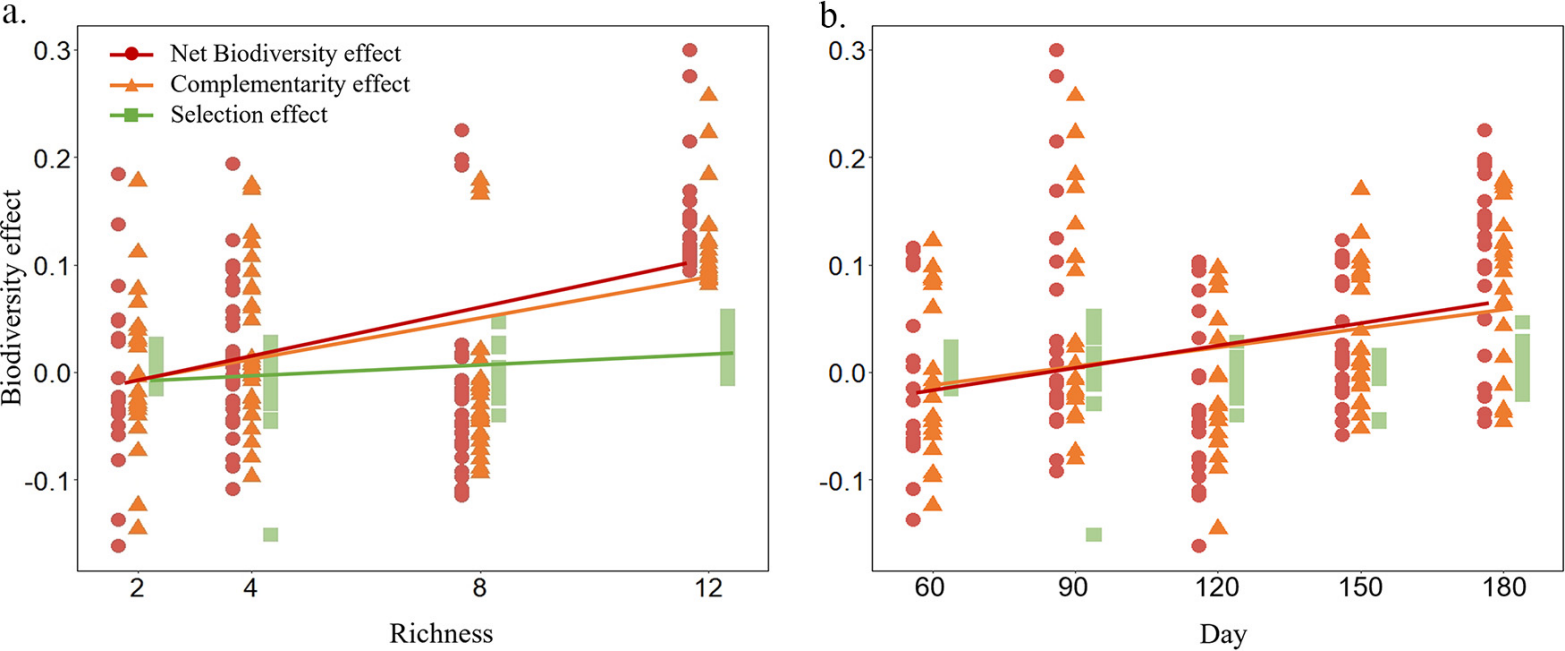
The overall relationship between biodiversity effects of richness (a) or time (b) on synthetic denitrifying communities. Significant relationships were observed for net biodiversity and richness [*F*_(1, 109)_ = 29.55; *P* < 0.001], complementarity effect and richness [*F*_(1, 109)_ = 22.62; *P* < 0.001], and selection effect and richness [*F*_(1, 109)_ = 18.12; *P* < 0.001]; significant relationships were also observed for biodiversity and time [*F*_(1, 109)_ = 10.88; *P = *0.001] and complementarity effect and time [*F*_(1, 109)_ = 10.14; *P* = 0.002]. Biodiversity effects were estimated by additive partitioning equation $\Delta Y=N\bar{\Delta \mathrm{RY}}\bar{N}+N\mathrm{cov}\left(\Delta \mathrm{RY},M\right)$, where Δ*Y* reflects net biodiversity effect, $N\bar{\Delta \mathrm{RY}}\bar{N}$ reflects complementarity effect, and Ncov⁡(ΔRY,M) reflects selection effect (49).

**FIG 5:**
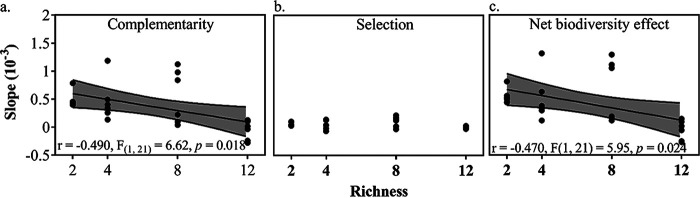
Relationships between richness and slopes of biodiversity effect versus time. (a) Complementarity. (b) Selection. (c) Net biodiversity effect.

To examine biodiversity effects on community functions along the evolution experiment, we partitioned the net biodiversity effect on productivity into complementarity and selection using an additive partitioning equation (see Fig. S5 in the supplemental material) (49). The results showed that overall complementarity (*P* < 0.001) and selection effects (*P* < 0.001) were significantly and positively correlated with richness, and the complementarity effect was generally larger than the selection effect (Fig. 4a). Also, complementarity significantly (*P* = 0.001) increased over time, while selection effect was similar over time (*P* = 0.079) along the evolution experiment (Fig. 4b). However, the increased degree of complementarity declined with the increasing richness of SDCs during the evolution experiment (Fig. 5), leading to significant negative relationships between richness and the slope of complementarity effect versus time (*P* = 0.018) or net biodiversity effect (*P* = 0.024), while no significant correlations were seen between richness and the slope of selection effect versus time (Fig. 5).

### Inferred species interactions during the evolution experiment.

**FIG 6:**
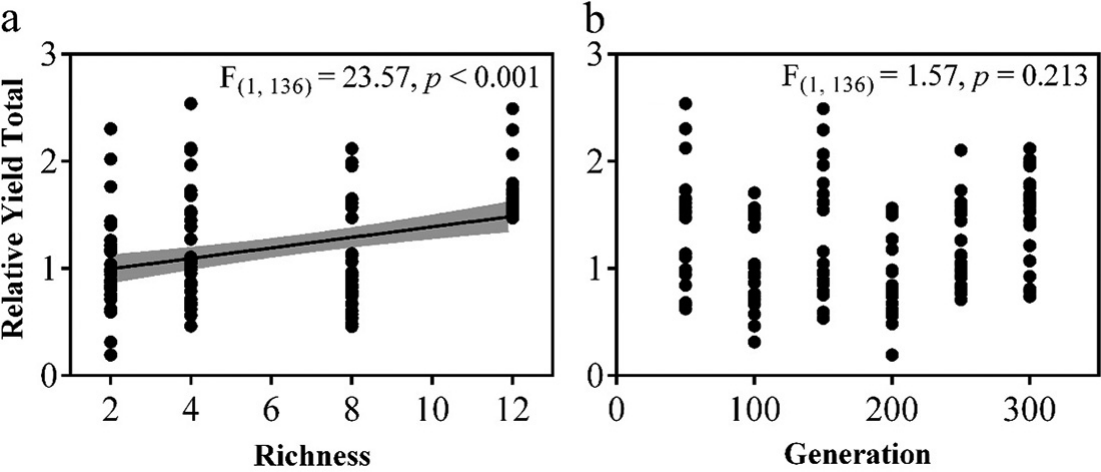
The relationship between the relative yield total of each community and richness (a) or time (b). The relative yield total (RYT) was calculated by summing the relative yield of all species in a mixture, representing the overall community interaction, and the relative yield (RY) was for each species in their corresponding monocultures.

To explore if the evolution of community functions is caused by alterations in microbial interaction patterns, we further inferred species interactions by comparing the contribution of each species in their mix-cultures and their corresponding monocultures (50, 51). Our results showed that the relative yield total was significantly (*P* < 0.001) positively correlated with richness (Fig. 6a). For each species, *S. marisflavi* EP1 presented the strongest positive average relative yield, while others were negative with time (see Fig. S6 in the supplemental material). Specifically, when the impact of time was only considered, the relative yield of S. putrefaciens CN-32 increased, while the relative yield of S. frigidimarina NCIMB 400 and *Shewanella* sp. W3_18_1 declined along the evolution experiment (see Table S4 in the supplemental material). These results indicated different degrees of impact of species interactions on observed positive and dynamic BEF relationships.

## DISCUSSION

Environmental changes affect the diversity and ecosystem function of complex communities. Especially on a long timescale, both ecological responses, including diversity changes and phenotypic changes of constituent species (52, 53), and evolutionary responses, such as the evolution of biotic interactions and genetic changes of constituent species (42, 54) of communities to environmental changes, could alter BEF relationships. In shifting environments, these complex and unpredictable ecological and evolutionary responses of communities constrain the prediction of BEF relationships. Recently, substantial efforts have been dedicated to understanding the relationship between biodiversity and ecosystem functioning and its dynamics in plant and animal systems (32, 34, 49). In this study, a richness gradient of SDCs and laboratory evolution approaches were used to explore the biodiversity-ecosystem functioning relationship and its dynamics in microbial ecosystems. We found overall positive BEF relationships for SDCs largely due to high complementarity, and such a BEF relationship was dynamic during the evolution experiment. As the BEF relationship shifted from significantly positive BEF relationships at early stages to no detectable BEF relationships at later stages of the evolution experiment, this could be largely due to a greater increase rate of complementarity effect or net biodiversity effect on low richness SDCs. These results well support our hypotheses that positive and dynamic biodiversity-ecosystem functioning relationships would occur due to changes in biodiversity effects during the evolution experiment.

The use of synthetic microbial communities and laboratory evolution of microbial communities have a few advantages for understanding BEF relationships, dynamics, and underlying mechanisms. First, as synthetic microbial communities with known genome information and physiological characteristics have low complexity and high controllability (29, 31), they are suitable for studying BEF relationships, interspecies interactions, and evolution of microbial communities. Second, most studies on ecology theories and the evolution of microbial communities are related to focal species (55, 56), while the synthetic microbial community approach is able to explicitly track the dynamics of the whole community, facilitating our mechanistic understanding of ecology and evolution of microbial communities. Third, understanding the evolution of interspecies interactions in a microbial community is challenging; synthetic microbial communities and laboratory evolution are able to address such key scientific questions (57). Therefore, this study aimed to understand the BEF relationship, dynamics, and underlying mechanisms of SDCs with both synthetic microbial ecology and experimental evolution approaches.

Positive BEF relationships may be largely due to the net biodiversity effect, including the complementarity effect and selection effect (27, 58, 59). For example, a reduction of denitrifier diversity in soil decreased denitrification activity due to a low functional redundancy, while resource addition could enhance positive BEF relationships through a higher functional complementarity in more diverse communities, which was due to the better use of overall resources compared to less diverse communities (60). A previous study reported positive effects of species richness on community denitrification and CO_2_ production, which was attributed to a broader complementary niche in more diverse communities (61). Additionally, the remarkable anaerobic versatility of *Shewanella* species could use a variety of different electron donors (46, 47), which may result in niche complementarity due to their plasticity of resource use. In this study, our results showed an overall positive correlation between richness and function (productivity or denitrification) during the evolution experiment, which could be largely due to a greater complementarity in diverse SDCs. The results are consistent with previous studies, showing that complementarity effects played a positive role in the determination of BEF relationships (20), and this positive effect was mediated by metabolic complementarity (62, 63). *Shewanella* species have different carbon utilization capacities, and they can potentially use different portions of the available resource pool. For example, among 10 available carbon compounds, 1-richness SDCs could use seven of them; 2-richness SDCs could use eight of them; 4-, 8-, and 12-richness SDCs could use all of them (64–67). If this were true, resource utilization efficiency would be higher in more diverse SDCs, resulting in higher productivity and denitrification as we observed in this study.

Selection effects, whereby diverse communities are more likely to contain more productive species, could also contribute to positive BEF relationships (21, 22). A previous study suggested that highly performing species were the primary driver of selection effects in a synthetic decomposing microbial community, and they were a predominant contributor to a positive BEF relationship as well (21). Another study showed that selection effects were positively associated with increasing richness at the outset of the evolution experiment of a synthetic microbial community consisting of environmental bacteria and then attenuated over an experimental course, indicating that the selection of specific bacteria became less important along the evolution experiment (45). In this study, we observed an obvious ecological sorting that productive species with higher growth rates (e.g., *S. marisflavi* EP1 for 0.226 h^−1^, *S. loihica* PV-4 for 0.176 h^−1^, S. putrefaciens CN-32 for 0.177 h^−1^) became dominant, and less competitive species with lower growth rates (e.g., *S. sediminis* HAW-EB3, *S. halifaxensis* HAW-EB4) gradually became undetectable along the evolution experiment. One of the reasons is that a series of transfers could lose some low abundant species during an evolution experiment (68). Those productive species generally had a better performance in multispecies SDCs, suggesting that selection effects had a positive impact on community functioning, which is consistent with the results from most short-term biodiversity studies (21, 61, 69). Also, we found that selection effects played a minor role at later evolution experiment stages, which is consistent with previous studies, showing that complementarity became more important and the selection of specific species became less important to community functioning over time (45, 70). This less important role of the selection effect in the long term may be because the productivity of low-richness SDCs (especially in monoculture) is not associated with competitive ability, and poor competitors, such as Shewanella mangrovi YQH10, could achieve high monoculture biomass in long-term serial transferring. The increasing contribution of complementary effect in BEF relationships over the long term may be due to the higher resource utilization efficiency of diverse SDCs. Therefore, we found an overall positive BEF relationship between the richness and function of SDCs, largely due to the complementarity effect, and selection also played a role, especially at the early stages of the evolution experiment. These findings provide empirical evidence for our understanding of BEF relationships and underlying mechanisms in microbial systems.

Evolutionary processes are an important driver of microbial community functioning over a long time scale, thus affecting BEF relationships (53, 57). Previous studies indicated the evolution of interspecies interactions ranging from mutualism to competition had important consequences for community functioning like productivity (54, 57). These temporal dynamics of BEF relationships have been observed in plant communities (50, 71–73) and recently in microbial communities as well (45, 54). For example, a previous study found a flattened BEF relationship due to a shift from the use of labile substrates at an early succession to the use of recalcitrant substrates at a later succession (45). In this study, we observed an overall increased ecosystem function (productivity and denitrification) as a result of the increased complementarity of SDCs along the evolution experiment. However, as the degree of complementarity declined with the increasing richness of SDCs, the effect of evolution on low richness SDCs was higher than that on high richness SDCs, resulting in an unexpectedly dynamic BEF relationship over time. One of the possibilities could be stronger constraints on the species trait distribution of high richness SDCs due to species interactions and ecological filtering, which leaves adaptive evolution much less room to alter species traits (42, 44). Another possibility could be that the low variation of functions in high richness SDCs reflected high temporal stability as diverse communities represent a form of biological insurance against the loss or poor performance of selected species (74). Therefore, our findings indicated that biodiversity could stabilize community functioning and constrain species’ evolutionary responses.

Microorganisms could accumulate beneficial mutations to gain growth fitness along a long-term evolution (39, 41), thus increasing community productivity through the growth enhancement of constituent species or productive mutualisms for synthetic microbial communities (75). For example, Pseudomonas fluorescens evolved alone was fitter than P. fluorescens evolved along with Pseudomonas putida due to beneficial mutations of acetate scavenger gene *actP*, which were exclusively detected in single-evolved P. fluorescens clones (43), indicating that biodiversity could limit evolutionary responses, as beneficial mutations were negated in more complex communities. Additionally, this provides a possible genetic mechanism to support the hypothesis that adaptive evolution would occur less frequently in more diverse communities (42, 43). Also, this study hinted that the selection of *Shewanella* strains could influence the trajectory of BEF relationship dynamics. For example, under the experimental culture conditions, benthic species *S. sediminis* HAW-EB3 and *S. halifaxensis* HAW-EB4 with lower growth rates were undetectable at the later period, thus affecting the function of the relevant SDCs and BEF relationships. Additionally, species isolated from the same or similar habitats may be introduced to interpret the evolution of BEF relationships. Therefore, further studies should focus on the understanding of molecular mechanisms of asymmetrical complementary effects on the dynamic BEF relationships, such as beneficial mutation analysis and gene expression profiling along an evolution experiment.

**FIG 7:**
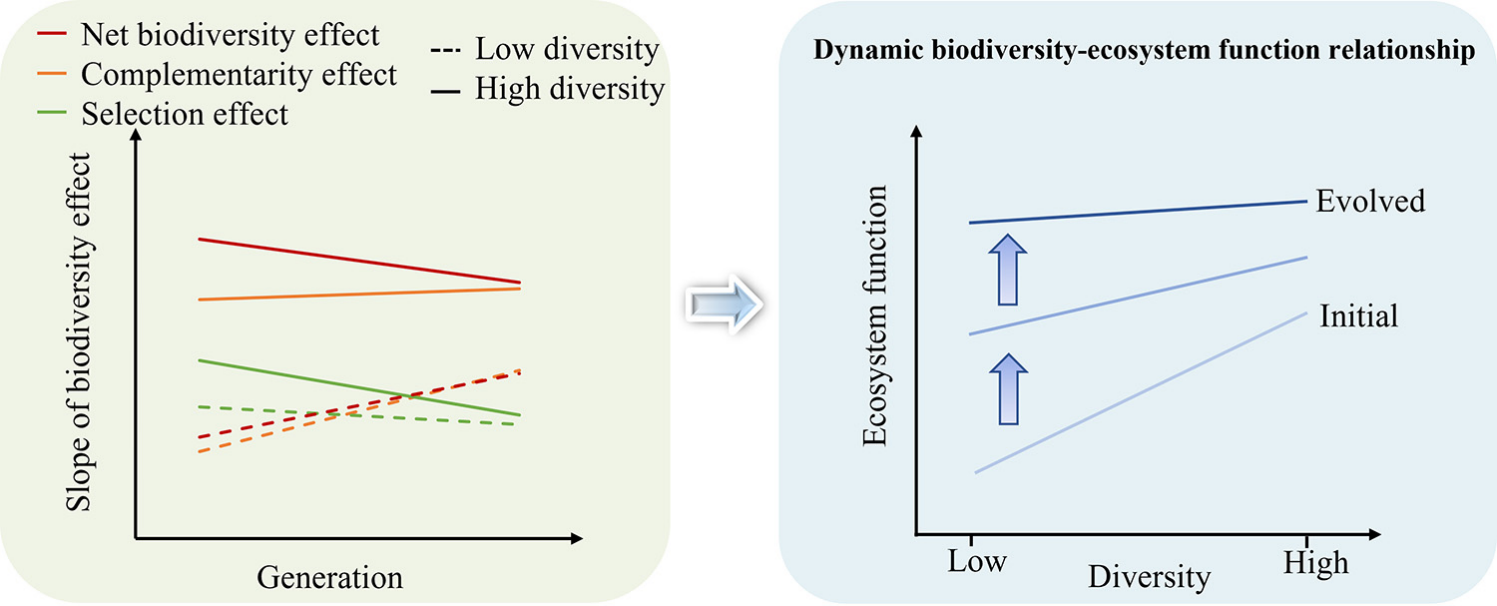
A schematic representation of possible mechanisms of biodiversity-ecosystem functioning relationships of synthetic microbial communities along the evolution experiment. A greater increase rate of complementarity and net biodiversity effects was observed at low-richness SDCs than at high-richness SDCs, and such asymmetrical changes of biodiversity effects could result in a flattened biodiversity-ecosystem functioning relationship in high-richness SDCs due to their lower increase rates along the evolution experiment. This less important role of selection effect along the long-term evolution experiment may be due to the increasing productivity of low-richness SDCs (especially the monoculture of poor competitors), which are less or not associated with competitive ability, while the increasing contribution of complementary effect in the biodiversity-ecosystem functioning relationship over the long-term may be due to the higher resource utilization efficiency of diverse SDCs.

In conclusion, we successfully constructed SDCs at a richness gradient of 1, 2, 4, 8, and 12 species with *Shewanella* denitrifiers. By analyzing their composition, productivity, and denitrification along an evolution experiment, we observed a positive BEF relationship, especially at the early stages of the evolution experiment. As a result of changes in composition, complementarity, and selection of SDCs along the evolution experiment, such a positive BEF relationship appeared to be dynamic (Fig. 7). This study advances our understanding of BEF relationships and their dynamics in microbial systems and provides new insights into biodiversity protection and microbial BEF relationship prediction.

## MATERIALS AND METHODS

### Selection of *Shewanella* denitrifiers and their growth conditions.

*Shewanella* bacteria are widely distributed among different habitats, such as sediment, seawater, and sea subsurface (47), and have special traits in nitrate respiration (46). Based on four criteria (the number of known denitrification genes in a genome, ≥2; the similarity of 16s rRNA genes in a genome, >95%; the genome similarity, >70%; the ability for partial or complete denitrification with the nitrate reductase gene as a prerequisite for synthetic denitrifying communities construction), we selected 12 *Shewanella* species/strains: S. decolorationis S12, S. putrefaciens CN-32, S. frigidimarina NCIMB 400, *Shewanella* sp. W3_18_1, *S. loihica* PV-4, *S. marisflavi* EP1, *S. mangrovi* YQH10, *S. amazonensis* SB2B, *S. fidelis* ATCC BAA-318, S. baltica OS155, *S. sediminis* HAW-EB3, and *S. halifaxensis* HAW-EB4 (see Table S1 in the supplemental material). The *Shewanella* species/strains were cultured anaerobically in three replicate Balch tubes at 25°C in the modified marine broth 2216 (Becton, Dickinson) supplemented with 20 mM NaNO_3_ as the electron acceptor.

### Synthetic denitrifying communities and biodiversity-ecosystem functioning relationships.

We constructed 30 synthetic denitrifying communities (SDCs) at a richness gradient of 1, 2, 4, 8, and 12 species with 6 replicates for each richness, and the composition of 1-, 2-, 4- and 8-species SDCs was created randomly (see Table S2 in the supplemental material). All 12 monocultures at the stationary growth phase were collected and measured using a hemocytometer, then all SDCs were inoculated with approximately the same total number of cells (1 × 10^8^ cells/mL), and the inocula in 2-, 4-, 8- and 12-species mixtures were equally mixed with corresponding species (Table S2). All SDCs were grown and propagated in the modified marine broth 2216 by transferring 3% (300 μL) of the final volume (10 mL) into a fresh medium after 72 h when all *Shewanella* bacteria reached their saturation stages, and each transfer was 3 days. This evolution experiment has been conducted for 180 days with 60 transfers.

We measured productivity and denitrification rates for all SDCs at the first transfer (3 days) and every 30 days (10 transfers, 1 month), respectively. The growth of all SDCs was monitored by a V-1000 spectrophotometer (AOE Instruments, China), and optical density at 600 nm (OD_600_) values of all SDCs were recorded every 2 to 3 h for 72 h, and community productivity was the maximum OD_600_. Also, we collected culture samples at the beginning (*t*_1_) and end (*t*_2_) of the log phase, and nitrate concentration (C) was measured using a San^++^ continuous flow analyzers (Skalar, Breda, Netherlands). The denitrification rate of SDCs was calculated as C(*t*_1_) − C(*t*_2_)/*t*_1_ − *t*_2_.

### Dynamics of SDC composition during evolution experiment.

To investigate the dynamics of SDC composition along evolution experiment, the abundance of individuals in SDCs was measured through quantitative PCR (qPCR). For the 3rd day and every 30 days, the genome DNA (gDNA) of SDCs at their maximum OD_600_ was extracted using a Rapid Bacterial Genomic DNA isolation kit (Sangon Biotech, Shanghai, China) following the manufacturer’s instructions. The quality of gDNA was assessed by NanoDrop ND-2000 spectrophotometer (Thermo Fisher Scientific, MA, USA) based on 260/230 and 260/280 ratios, and gDNA samples were then diluted into 1 to 10 ng/μL for subsequent qPCR quantification. Specific primers for each strain were designed in specific regions of single-copy genes (see Table S3 in the supplemental material) using *k*-mer approaches (76, 77). The specificity of each primer pair was checked using the NCBI Primer-BLAST (78) and then validated by PCR and gel electrophoresis. Purified PCR products of specific genes were cloned into T-vector (pEASY TA vector; TransGen, China) to construct plasmids for qPCR standard curves. The concentration of plasmids was determined by Qubit 4.0 (Thermo Fisher Scientific, MA, USA). The qPCR was carried out in a 12-μL reaction system containing 6 μL iTaq Universal SYBR green supermix (Bio-Rad laboratories, Hercules, CA, USA), 0.5 μL of each 10-mM forward and reverse primers, and 3.8 μL nuclease-free water. Standard and DNA samples were added at 1.2 μL per reaction. The reaction was carried out in triplicate on a CFX96 real-time system (Bio-Rad Laboratories, Hercules, CA, USA) using the following program: 95°C for 4 min followed by 40 cycles of 95°C for 30 s, 62 to 65°C for 30 s, and 72°C for 20 s. Melting curve analyses (cooling amplified products to 65°C and then gradually heating them to 95°C at a rate of 0.2°C/s) and gel electrophoresis were performed to confirm that the amplified products were of the appropriate site. Copy numbers of the *Shewanella* species were analyzed using a regression equation for each assay relating the cycle threshold (*C_T_*) value to the known number of copies in the standards. The amplification efficiency of qPCR ranged from 90% to 110%, and the correlation coefficient (*r*^2^) of standard curves was greater than 0.98.

### Estimation of biodiversity effects and species interactions.

The biodiversity effects, including complementarity and selection effect, were estimated as the difference between observed and expected productivity by additive partitioning equation (49) as follows:

$$\Delta Y=Y_{O}-Y_{E}=\sum_{i}RY_{O,i}M_{i}-\sum_{i}RY_{E,i}M_{i}=\sum_{i}\Delta RY_{i}M_{i}=N\bar{\Delta \mathrm{RY}}\bar{M}+N\mathrm{cov}(\Delta \mathrm{RY},M),$$

where Δ*Y* = *Y_O_* − *Y_E_*, is the difference between the observed and expected function of SDCs, *Y_O_* = $\sum_{i}Y_{O,i}=\sum_{i}RY_{O,i}M_{i}$ (total observed yield of the mix-culture), $Y_{E}=\sum_{i}Y_{E,i}=\sum_{i}RY_{E,i}M_{i}$ (total expected yield of the mix-culture), *N* is the number of species in the SDC, Δ*RY* is the difference between the function of proportion seeded of a particular species in a monoculture and in a corresponding mix-culture, and *M_i_* is the yield of species *i* of monoculture. In the equation, Δ*Y* reflects net biodiversity effect, $N\bar{\Delta \mathrm{RY}}\bar{M}$ reflects complementarity effects, and they measure the change in the average relative yield in the mix-culture. Ncov⁡(ΔRY,M) reflects selection effects and is measured by a covariance function based on Price’s general theory of selection (49, 79). The relative yield total (RYT) measures the overyield by summing the relative yields of all species in a mix-culture, and an RYT of >1 indicates complementarity (50). The relative yield of a species (RY) represents the impact of given populations on the function attributed to a particular species and was calculated by dividing its biomass in a mix-culture by its monoculture biomass (51).

### Statistical analysis.

We used the Spearman correlation (lm in R 4.0.1) to calculate ecosystem functions (productivity/denitrification rate) and richness/time relationships and fitted the model using the stepAIC function in MASS. General linear models (GLMs) and Spearman’s correlation were used to evaluate the effect of biodiversity and evolution time on community functions.

### Data availability.

Data are provided as private-for-peer review and shared publicly on https://osf.io/kgq27/?view_only=d5781ef86cbb4aed839cc93dcb338148.
