# Supplementary material for: Synthetic Denitrifying Communities Reveal a Positive and Dynamic Biodiversity-Ecosystem Functioning Relationship during Experimental Evolution
Source: Microbiol Spectr. 2023 May 8;11(3):e04528-22. doi: 10.1128/spectrum.04528-22 (PMC10269844; doi:10.1128/spectrum.04528-22)
Supplement: Supplemental file 1 — Supplemental material. Download spectrum.04528-22-s0001.pdf, PDF file, 0.9 MB [file spectrum.04528-22-s0001.pdf]

Manuscript title:

Synthetic denitrifying communities reveal a positive and dynamic biodiversity-ecosystem functioning relationship during experimental evolution

Author list:

Bo Wu[1], Xiaotong Guan[1], Ting Deng[1], Xueqin Yang[1], Juan Li[2], Min Zhou[1], Cheng Wang[1], Shanquan Wang[1], Qingyun Yan[1], Longfei Shu[1], Qiang He[3] and Zhili He[1, 2*]

[1]Environmental Microbiomics Research Center, School of Environmental Science and Engineering, Southern Marine Science and Engineering Guangdong Laboratory (Zhuhai), Sun Yat-sen University, Guangzhou 510006, China; [2]College of Agronomy, Hunan Agricultural University, Changsha 410128, China; [3]Department of Civil and Environmental Engineering, the University of Tennessee, Knoxville, TN 37996, USA

[*]**Corresponding author**: Zhili He

132 East Circle, University Town, Guangzhou 510006, China

Phone: +86-20-3106-5837

Email: hezhili@mail.sysu.edu.cn

Supplementary figures

Figure S1 Growth of 12 *Shewanella* strains in a modified 2216 medium

Figure S2 Relationships between time and function (productivity/denitrification rate) or between richness and slopes of functions vs. time of synthetic denitrifying communities

Figure S3 Temporal dynamics of synthetic denitrifying community composition during the evolution experiment

Figure S4 Relative abundances of each community composition at different richness levels

Figure S5 The role of biodiversity effects on synthetic denitrifying communities during the evolution experiment at (a) 30th, (b) 60th, (c)90th, (d) 120th, (e) 150th and (f) 180th days

Figure S6 Overall relative yield of *Shewanella* species.

Supplementary tables

Table S1 Information and characteristics of *Shewanella* species/strains and denitrification genes used in this study

Table S2 The composition of synthetic denitrifying communities at different richness levels

Table S3 Specific primers for quantitative PCR analysis of abundances of *Shewanella* species/strains in this study

Table S4 The linear regression and correlation between each species interaction relative yield and time

Figure S1 Growth of 12 *Shewanella* strains in a modified 2216 medium. All the Shewanella strains were grown separately in a modified marine broth 2216 (Becton Dickinson) anaerobically in Balch tubes at 25°C and supplemented with 20 mM NaNO3 as the electron acceptor.

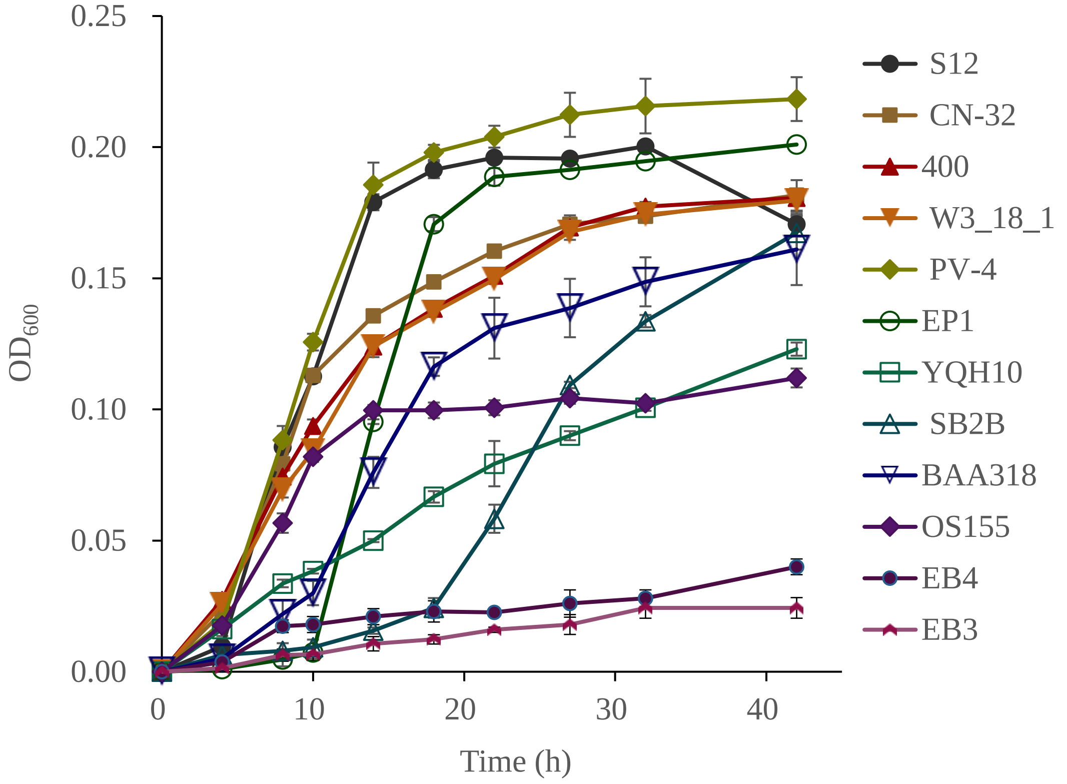

Figure S2 Relationships between time and function (productivity/denitrification rate) or between richness and slopes of functions vs. time of synthetic denitrifying communities. a. the overall relationship between productivity and evolution time; b. the overall relationship between denitrification and evolution time; c. Significant relationships were observed between productivity and time at the richness of 1 (F(1, 40) = 31.21, *p* <0.001); d. Relationships between denitrification and time were significant at the richness of 1 (F(1, 40) = 17.07, *p* <0.001), 2 (F(1, 40) = 9.46, *p* = 0.004), and 4 (F(1, 40) = 10.96, *p* = 0.002); e. A negative relationship between richness and the slope of productivity/time (slope = -0.345, $F_{(1, 28)}$ = 10.95, *p* = 0.003); f. A negative relationship between richness and the slope of denitrification/time (slope = -0.087, $F_{(1, 28)}$ = 4.54, *p* = 0.042).

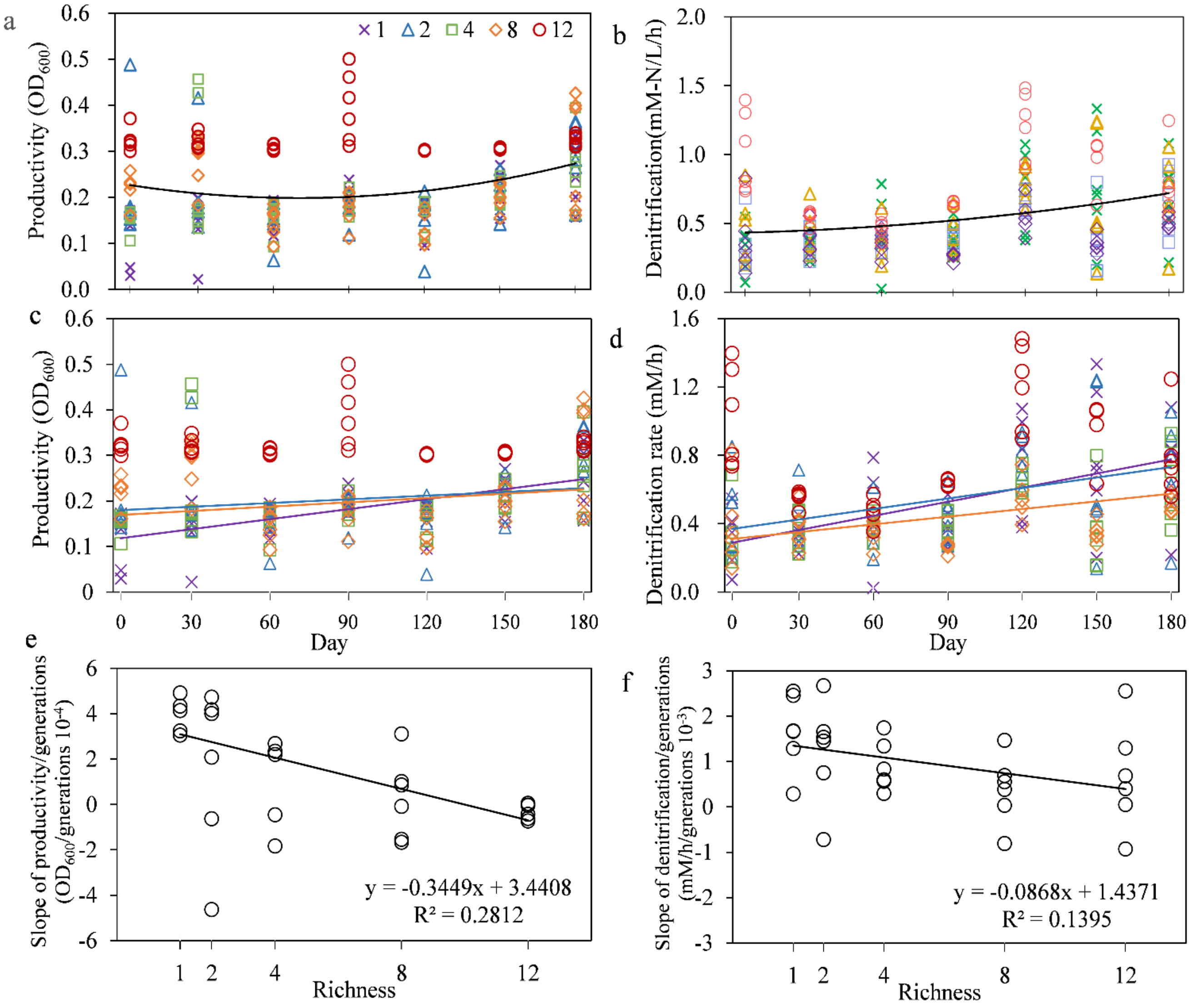

Figure S3 Temporal dynamics of synthetic denitrifying community composition during the evolution experiment. Log-transferred absolute abundances of individual species/strains in their communities were shown with six replicates for each richness SDCs. (a) 1-species SDCs; (b) 2-species SDCs; (c) 4-species SDCs; (d) 8-species SDCs. Species abbreviations: S12, *Shewanella decolorationis* S12; CN-32, *S. putrefaciens* CN-32; 400, *S. frigidimarina* NCIMB 400; W3_18_1, *Shewanella* sp. W3_18_1; PV-4, *S. loihica* PV-4; EP1, *S. marisflavi* EP1; YQH10, *S. mangrovi* YQH10; SB2B, *S. amazonensis* SB2B; BAA-318, *S. fidelis* ATCC BAA-318; OS155, *S. baltica* OS155; HAW-EB3, *S. sediminis* HAW-EB3; HAW-EB4, *S. halifaxensis* HAW-EB4.

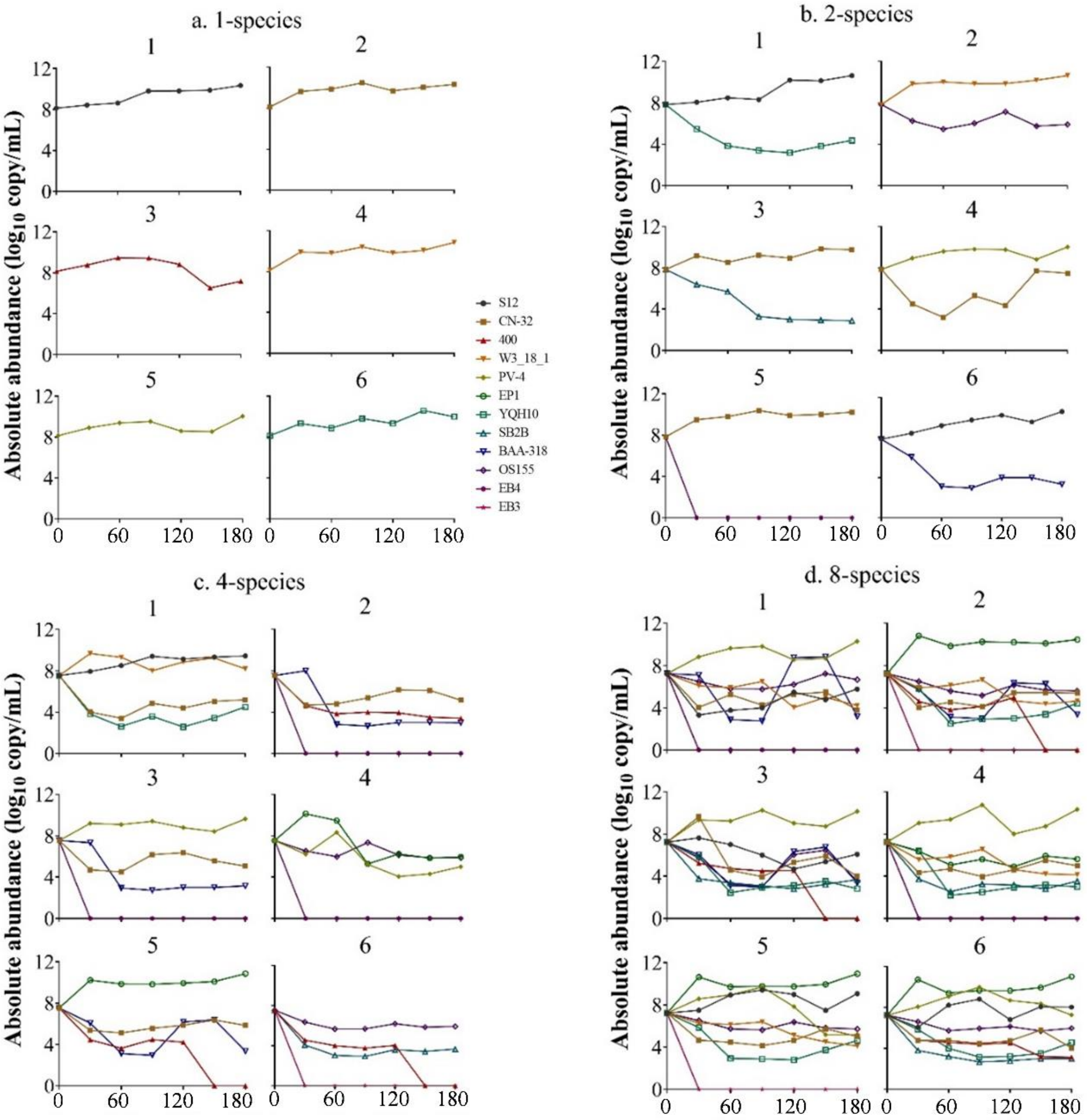

Figure S4 Relative abundances of each community composition at different richness levels. We did not show the relative abundance of *S. sediminis* HAW-EB3 and *S. halifaxensis* HAW-EB4 as they were undetectable. A black line was generated by linear regression between observed relative abundances and richness with significance at $p < 0.05$, while a dashed line was generated by linear regression between expected relative abundances and richness with an expected relative abundance of 0.5, 0.25, 0.125 and 0.083 for each species in 2-, 4-, 8- and 12-species SDCs, respectively.

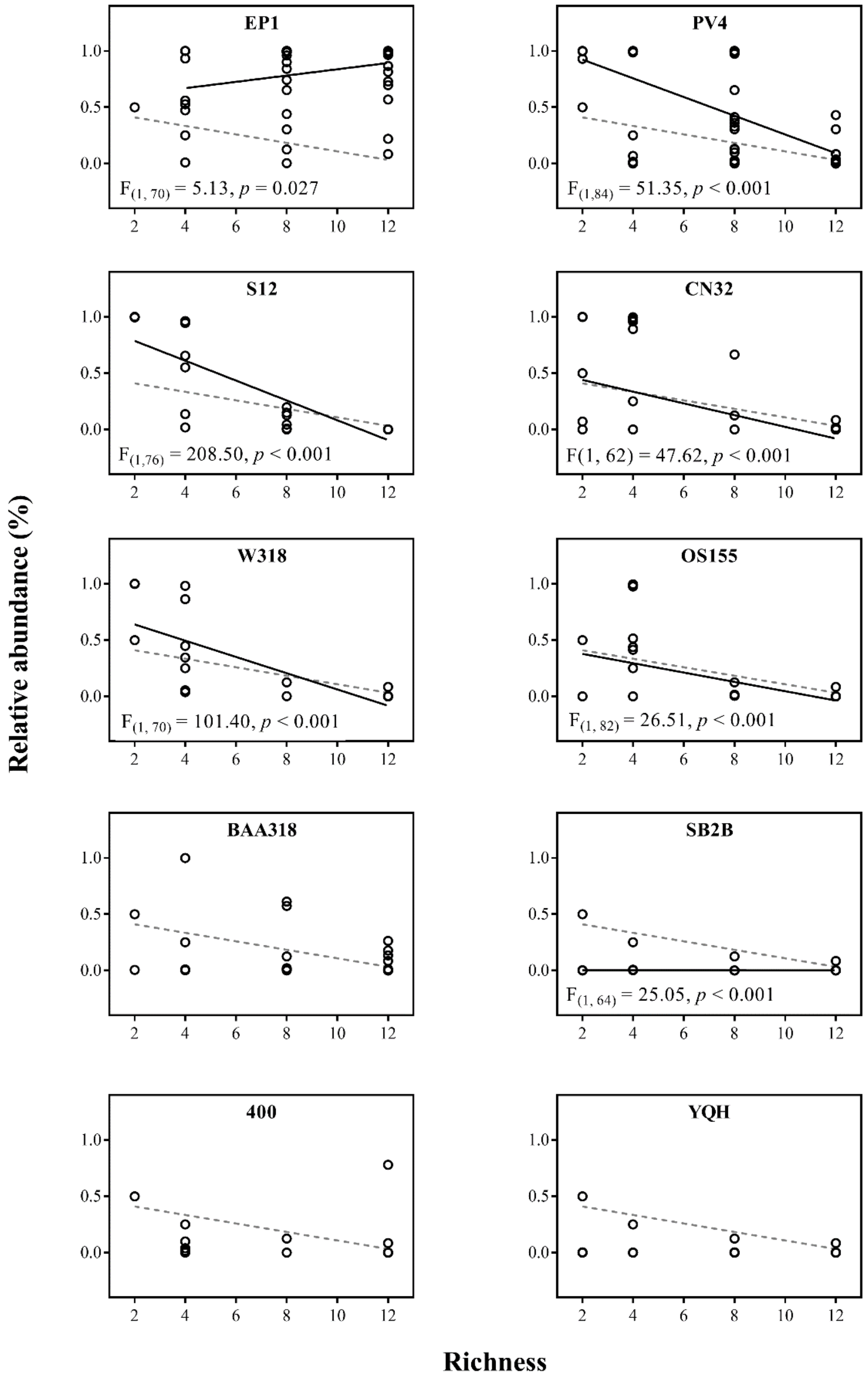

Figure S5 The role of biodiversity effects on SDCs during the evolution experiment at (a) 30th, (b) 60th, (c)90th, (d) 120th, (e) 150th, and (f) 180th days. Biodiversity effects were estimated by additive partitioning equation $\Delta Y = N\overline{\Delta RY}\,\overline{N} + N\,\mathrm{cov}(\Delta RY, M)$, where ΔY reflects net biodiversity effect, $N\overline{\Delta RY}\,\overline{N}$ reflects the complementarity effect, and $N\,\mathrm{cov}(\Delta RY, M)$ reflects the selection effect.

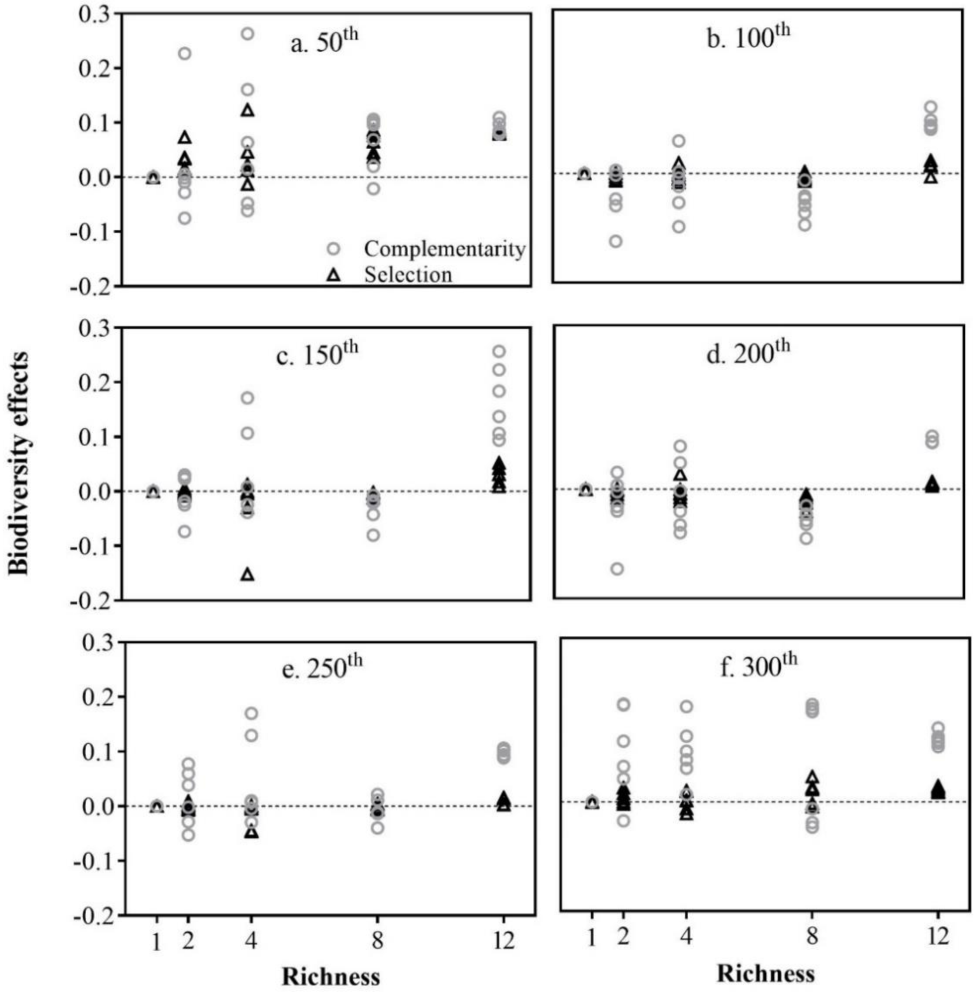

Figure S6 Overall relative yield of the *Shewanella* species. The relative yield of specific species was calculated by comparing the contribution of each species in their SDCs to their monocultures.

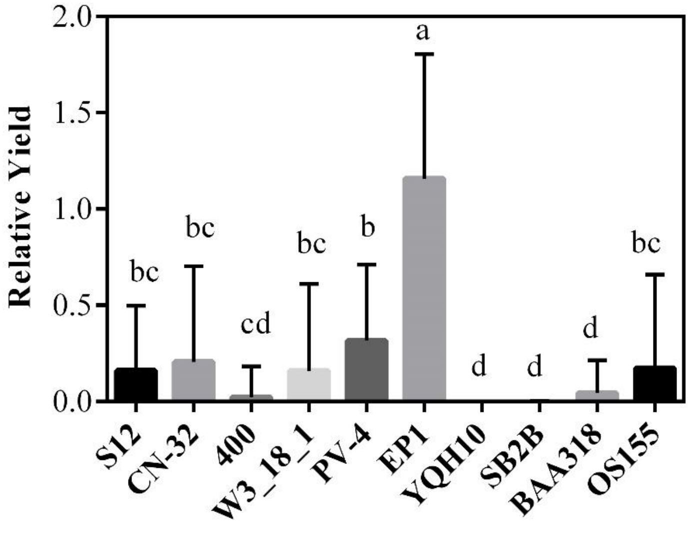

Table S1 Information and characteristics of *Shewanella* species/strains and denitrification genes used in this study.

	S12	CN-32	400	W3_18_1	PV-4	EP1	YQH10	SB2B	BAA-318	OS155	EB3	EB4
Accession No.	AXZL01000000	CP000681	NC_008345	KI912458	NC_008700	NZ_CP022272	JPEO01000001	NC_008700	KI912458.1	CP000563	KI912458	NC_010334
nar	+	+	+	+	+	+	+	+	+	+	+	+
nir	+	+	+	+	+	+	+	+	+	+	+	+
nor	-	+	+	+	+	+	-	+	-	-	+	+
nos	-	-	-	-	+	-	-	-	-	-	-	-
Habitat	Activated sludge	Shale sandstone	Sea water	Marine sediments	Microbial mat	Marine sediments	Mangrove sediment	Marine sediments	Marine sediments	Marine sediments	Harbor sediment	Marine sediment
Growth pH	8	7-8	6.2-6.5	7.0	6-8	7-8	6	7-8	7.5	8	7.3	7.3
Carbon Utilization												
Glucose	+	-	+	-	+	+	+	-	+	+	-	+
Citrate	-	-	+	+	+	+	-	+	-	+	-	-
Lactate	+	+	+	+	+	+	NA	+	-	+	+	+
Succinate	+	+	+	+	+	+	+	+	-	-	+	-
Sucrose	+	+	+	-	-	-	NA	-	-	+	-	-
Fumarate	-	+	+	+	+	+	NA	+	-	-	NA	-
Pyruvate	+	NA	+	+	+	NA	+	-	NA	+	+	+
Cellobiose	+	-	+	-	-	+	+	NA	-	+	-	-
Acetate	+	+	+	+	-	NA	NA	+	-	NA	+	+
Fructose	+	+	+	-	NA	-	+	+	-	NA	-	-
Reference	[1, 2]	[3]	[4] [5]	[3]	[6]	[7, 8]	[9]	[6, 10]	[11]	[12]	[13]	[14]

Notes: +, positive; -, negative; NA, no data. Species abbreviations are as follows: S12, *Shewanella decolorationis* S12; CN-32, *Shewanella putrefaciens* CN-32; 400, *Shewanella frigidimarina* NCIMB 400; W3_18_1, *Shewanella* sp. W3_18_1; PV-4, *Shewanella loihica* PV-4; EP1, *Shewanella marisflavi* EP1; YQH10, *Shewanella mangrovi* YQH10; SB2B, *Shewanella amazonensis* SB2B; BAA-318, *Shewanella fidelis* ATCC BAA-318; OS155, *Shewanella baltica* OS155; HAW-EB3, *Shewanella sediminis* HAW-EB3; HAW-EB4, *Shewanella halifaxensis* HAW-EB4. Gene information is based on http://www.microbesonline.org/ and https://img.jgi.doe.gov/.

Table S2 The composition of synthetic denitrifying communities at different richness levels.

	1-species						2-species						4-species						8-species						12-species					
Strain	1	2	3	4	5	6	1	2	3	4	5	6	1	2	3	4	5	6	1	2	3	4	5	6	1	2	3	4	5	6
S12	■						■					■	■						■		■		■	■	■	■	■	■	■	■
CN-32		■							■	■	■		■	■	■		■		■	■	■	■	■	■	■	■	■	■	■	■
400			■											■			■	■		■	■			■	■	■	■	■	■	■
W3_18_1				■				■					■						■	■		■	■		■	■	■	■	■	■
PV-4					■					■					■	■			■		■	■	■	■	■	■	■	■	■	■
EP1																■	■			■		■	■	■	■	■	■	■	■	■
YQH10						■	■						■							■	■	■	■	■	■	■	■	■	■	■
SB2B									■									■			■	■		■	■	■	■	■	■	■
BAA-318												■		■	■		■		■	■	■				■	■	■	■	■	■
OS155								■								■		■	■	■	■		■	■	■	■	■	■	■	■
HAW-EB4											■			■	■	■			■			■			■	■	■	■	■	■
HAW-EB3																		■	■	■		■	■		■	■	■	■	■	■

Notes: Species abbreviations are as follows: S12, *Shewanella decolorationis* S12; CN-32, *Shewanella putrefaciens* CN-32; 400, *Shewanella frigidimarina* NCIMB 400; W3_18_1, *Shewanella* sp. W3_18_1; PV-4, *Shewanella loihica* PV-4; EP1, *Shewanella marisflavi* EP1; YQH10, *Shewanella mangrovi* YQH10; SB2B, *Shewanella amazonensis* SB2B; BAA-318, *Shewanella fidelis* ATCC BAA-318; OS155, *Shewanella baltica* OS155; HAW-EB3, *Shewanella sediminis* HAW-EB3; HAW-EB4, *Shewanella halifaxensis* HAW-EB4.

Table S3 Specific primers for quantitative PCR analysis of abundances of *Shewanella* species/strains in this study.

Strain	Forward (5'-3')	Reverse (5'-3')
Shewanella decolorationis S12	CCCGACACCTTAACCTATGAATCT	GCTACCCTCGGCTCGACAAT
Shewanella putrefaciens CN-32	AACACTTGCCTTGAGTTACCT	TCAATGATTCTATCCGTGGTGT
Shewanella frigidimarina NCIMB 400	ACTCGGTTAGAGTGCAGACTTC	CTGCTTGTTCTGAATGAACTTGTC
Shewanella sp. W3_18_1	TGCCCATCTCAAACGCAAATC	GAAGATGCCATGACAATCAAGGTC
Shewanella loihica PV-4	CACCACTATCCGACAGCCAT	ATCCCAGACCGTACCACCAT
Shewanella marisflavi EP1	TGCCTGGGCGAATGTTAGAC	GGCGTAACGCACTAATTCCT
Shewanella mangrovi YQH10	GCCATTCATACTTGCCGTTGG	CGAGCAGTTTATTCGTAGTCATCC
Shewanella halifaxensis HAW-EB4	GCTGCTAGCTAATGATGAAGCC	ACAAGTACCACTTCTTGCCGT
Shewanella amazonensis SB2B	GCTGGTATCACGTCAACTGGT	TGTCTGCTTACCGGCTTTGA
Shewanella fidelis ATCC BAA-318	TTGCGCGACCGTTAGTATTG	TTTGGCATCCACAATCCTGC
Shewanella baltica OS155	AAGCCATTTGACTCTGACGGT	CAGATCCTGGCAGTTGGGTA
Shewanella sediminis HAW-EB3	GGTTTGAGTTGCCTTGAGCGTA	TTGTGGCAGATGAAGTGCGTC

Table S4 The linear regression between each species relative yield and time. The *p* values were based on the Spearman correction analysis.

	S12	CN-32	400	W_3_18_1	PV-4	EP1	YQH10	SB2B	BAA-318	OS155
Slope	4.51×10^{-4}	2.60×10^{-4}	-2.09×10^{-4}	-4.22×10^{-4}	9.21×10^{-5}	6.60×10^{-4}	-1.95×10^{-6}	-8.07×10^{-7}	-1.27×10^{-5}	3.54×10^{-4}
Spearman *r*	0.088	0.258	-0.494	-0.532	-0.032	0.103	-0.002	-0.110	-0.120	-0.058
p value	0.444	0.006	<0.001	<0.001	0.771	0.392	0.988	0.381	0.296	0.601

References

1. Xu M, Guo J, Cen Y, Zhong X, Cao W, Sun G. Shewanella decolorationis sp. nov., a dye-decolorizing bacterium isolated from activated sludge of a waste-water treatment plant. Int J Syst Evol Microbiol. 2005; **55:** 363-368.
2. Hong Y, Xu M, Guo J, Xu Z, Chen X, Sun G. Respiration and growth of Shewanella decolorationis S12 with an Azo compound as the sole electron acceptor. Appl Environ Microbiol. 2007; **73:** 64-72.
3. Qiu D, Tu Q, He Z, Zhou J. Comparative Genomics Analysis and Phenotypic Characterization of Shewanella putrefaciens W3-18-1: Anaerobic Respiration, Bacterial Microcompartments, and Lateral Flagella. Lawrence Berkeley National Laboratory. 2010.
4. Gordon EH, Pike AD, Hill AE, Cuthbertson PM, Chapman SK, Reid GA. Identification and characterization of a novel cytochrome c(3) from Shewanella frigidimarina that is involved in Fe(III) respiration. Biochem J. 2000; **349:** 153-158.
5. Reid GA, Gordon EHJ. Phylogeny of marine and freshwater Shewanella: reclassification of Shewanella putrefaciens NCIMB 400 as Shewanella frigidimarina. International Journal of Systematic and Evolutionary Microbiology. 1999; **49:** 189-191.
6. Karpinets TV, Obraztsova A, Wang Y, Schmoyer D, Kora G, Park BH et al. Conserved synteny at the protein family level reveals genes underlying Shewanella species' cold tolerance and predicts their novel phenotypes. PLoS Comput Biol. 2010; **10**.
7. Huang J, Ning G, Li F, Sheng GD. Biotransformation of 2,4-dinitrotoluene by obligate marine Shewanella marisflavi EP1 under

anaerobic conditions. Bioresource Technology. 2015; **180:** 200-206.

8. Huang J, Sun B, Zhang X. Electricity generation at high ionic strength in microbial fuel cell by a newly isolated Shewanella marisflavi EP1. Appl Microbiol Biot. 2010; **85:** 1141-1149.

9. Liu Y, Shang XX, Yi ZW, Gu L, Zeng RY. Shewanella mangrovi sp. nov., an acetaldehyde-degrading bacterium isolated from mangrove sediment. Int J Syst Evol Microbiol. 2015; **65:** 2630-2634.

10. Venkateswaran K, Dollhopf ME, Aller R, Stackebrandt E, Nealson KH. Shewanella amazonensis sp. nov., a novel metal-reducing facultative anaerobe from Amazonian shelf muds. Int J Syst Evol Microbiol. 1998; **48:** 965-972.

11. Ivanova EP, Sawabe T, Hayashi K, Gorshkova NM, Zhukova NV, Nedashkovskaya OI et al. Shewanella fidelis sp. nov., isolated from sediments and sea water. International Journal of Systematic and Evolutionary Microbiology. 2003; **53:** 577-582.

12. Kan J, Flood BE, Mccrow JP, Kim JS, Tan L, Nealson KH. A rapid fingerprinting approach to distinguish between closely related strains of Shewanella. J Microbiol Methods. 2011; **86:** 62-68.

13. Zhao JS, Manno D, Beaulieu C, Paquet L, Hawari J. Shewanella sediminis sp. nov., a novel Na+-requiring and hexahydro-1,3,5-trinitro-1,3,5-triazine-degrading bacterium from marine sediment. Int J Syst Evol Microbiol. 2005; **55:** 1511-1520.

14. Zhao J, Manno D, Leggiadro C, Oneil D, Hawari J. Shewanella halifaxensis sp. nov., a novel obligately respiratory and denitrifying psychrophile. Int J Syst Evol Microbiol. 2006; **56:** 205-212.
